# Adolescents with Persistent Symptoms Following Acute SARS-CoV-2 Infection (Long-COVID): Symptom Profile, Clustering and Follow-Up Symptom Evaluation

**DOI:** 10.3390/children12010028

**Published:** 2024-12-27

**Authors:** Marco Floridia, Danilo Buonsenso, Laura Macculi, Liliana Elena Weimer, Marina Giuliano, Flavia Pricci, Leila Bianchi, Domenico Maurizio Toraldo, Graziano Onder, The ISS Long-COVID Study Group

**Affiliations:** 1 National Center for Global Health, Istituto Superiore di Sanità, 00161 Rome, Italy; liliana.weimer@iss.it (L.E.W.); marina.giuliano@iss.it (M.G.); 2 Department of Woman and Child Health and Public Health, Fondazione Policlinico Universitario A. Gemelli IRCCS, 00136 Rome, Italy; 3 Fondazione Policlinico Universitario A. Gemelli IRCCS, Università Cattolica del Sacro Cuore, 00136 Rome, Italygraziano.onder@unicatt.it (G.O.); 4 Department of Cardiovascular, Endocrine-Metabolic Diseases and Ageing, Istituto Superiore di Sanità, 00161 Rome, Italy; 5 Infectious Diseases Unit, Meyer Children’s Hospital IRCCS, 50139 Florence, Italy; leila.bianchi@meyer.it; 6 Cardiorespiratory Rehabilitation Unit, Department of Rehabilitation, “V. Fazzi” Hospital, 73100 Lecce, Italy; 7 Office of the President, Istituto Superiore di Sanità, 00161 Rome, Italy

**Keywords:** COVID-19, long-COVID, post-COVID, symptoms, symptom clusters, adolescents, fatigue, dyspnea

## Abstract

Background: Few studies have evaluated long-COVID in adolescents. Methods: Cohort study. Demographics, clinical data, and the presence of 30 symptoms were collected with a modified WHO form. Mean values were compared by Student’s *t* test and proportions by the chi-square test or Fisher test, with trends over time analysed using the chi-square test for trend. Potential risk factors independently associated with persisting symptoms were evaluated in a multivariable logistic regression model. Clustering of cases was analysed by two-step automatic clustering. Results: A total of 97 adolescents aged 12–17 (54.6% females, 45.4% males) were evaluated. After a mean interval of 96 days (SD 52) from acute infection, the mean number of symptoms (2.8 overall) was higher for pre-Omicron (3.2 vs. 2.5 in Omicron, *p* = 0.046) and moderate/severe acute infections (4.2 vs. 2.7 in mild, *p* = 0.023). Fatigue (62.9%) and dyspnea (43.3%) were the most common symptoms, followed by headache (28.9%), thoracic pain (22.7%), diarrhea (20.6%), palpitations/tachycardia (17.5%), articular pain (15.5%), difficult concentration (14.4%), muscle pain (12.4%), taste reduction (8.2%), smell reduction (8.2%), fever (6.2%), and skin disorders (5.2%). The symptom profile was similar in males and females but showed significant differences from that observed in concurrently followed adults. After a mean interval of 340 days from infection, 45.3% still presented symptoms, with persistence associated with higher number of initial symptoms. Two clusters were defined that differed in the phase of acute infection and the number and profile of symptoms. Conclusions: Long-COVID manifestations in adolescents may differ from those observed in adults. Polisymptomaticity may predict long-term persistence.

## 1. Introduction

The long-term sequelae of COVID-19 infection are increasingly recognised as a significant cause of morbidity and disability worldwide [1,2,3]. The UK National Institute for Health and Care Excellence (NICE) defines ‘Long-COVID’ as any symptom persistence beyond four weeks [4], in concordance with the definition of the US Department of Health and Human Services (DHHS) and the Centers for Diseases Control and Prevention (CDC) [5]. The WHO has defined post COVID-19 condition/long COVID as the continuation or development of new symptoms three months after the initial SARS-CoV-2 infection [6], and subsequently issued a separate clinical case definition for children and adolescents [7], acknowledging that symptoms in children and adolescents are non-specific and can occur with other infections and illnesses, complicating the diagnosis. Such difficulties, together with the variable definitions used in clinical studies, have led to a wide array of the prevalence of paediatric long-COVID in the scientific literature. In some studies, prevalence has peaked with frequencies of 20% or higher [8,9,10,11,12,13]. More recent studies have suggested lower rates, with 1–5% of children and adolescents affected [10,14]. The prevalence of individual symptoms is also ill-defined and similarly variable [15,16]; only a few studies have compared the symptom profiles in contemporary enrolled adults and paediatric populations [17]. Finally, the vast majority of the studies conducted in younger individuals have evaluated mixed populations of children and adolescents, who may, however, differ significantly in several aspects.

With the aim to contribute information on this issue, we used data from a multicentre national cohort to define the clinical characteristics of adolescent patients accessing care for long-COVID in specialised centres, the profile of symptoms reported, their persistence during follow-up, and the possible clustering of cases.

## 2. Materials and Methods

The present clinical study is part of the larger project “Analysis and strategies for responding to the long-term effects of the COVID-19 infection (Long-COVID)”, funded by the National Centre for Disease Prevention and Control (CCM) of the Italian Ministry of Health. Within this project, before the current study, a national survey was conducted to identify centres providing care to individuals with long-COVID [18].

The project, patient information sheet, and consent form were approved by the Italian National Ethics Committee (AOO-ISS—19/04/2022–0015066 Class: PRE BIO CE 01.00). Written informed consent was required for patient inclusion. This study started in January 2023 and was closed in March 2024, with data extracted on 2 April 2024. Data were entered by medical staff in participating centres using an online dedicated platform with two levels of authentication that included one-time passwords. For data collection, a shortened version of the Post COVID-19 CRF from the WHO Global Clinical Platform for COVID-19 was used, that included demographics, comorbidities, severity, and timing of acute COVID, subjective functional status after infection, plus 30 different symptoms and the presence of 34 specific clinical diagnoses [19]. Adolescents underwent two clinical evaluations using the above form for data collection, with the first evaluation conducted at least four weeks after acute infection (recommended: between 3 and 6 months) and the second (follow up) after at least three months from first visit (recommended: 6–12 months from acute infection).

Inclusion criteria were age between 12 and 17 years, known date of acute SARS-CoV-2 infection, and at least one symptom persisting for more than four weeks after acute infection. Severity of acute SARS-CoV-2 disease was defined as mild, moderate, severe, or critical according to the WHO grading [19]. Respiratory assistance was categorised as none, low, or high flow oxygen, continuous positive airway pressure (CPAP), mechanical ventilation, and extracorporeal membrane oxygenation (ECMO). The phase of the pandemic was categorised as pre-Omicron or Omicron according to the occurrence of date of acute infection before or after 23 December 2021 [20]. The prevalence of symptoms was compared with that observed among adult patients contemporarily enrolled in the same project [21].

Data were summarised as a proportion, for categorical variables, and as a mean with standard deviation, for quantitative variables. Mean values were compared by Student’s *t* test and proportions by the chi-square test or Fisher test in contingency tables. Trends over time in proportions were analysed using the chi-square test for trend.

Clustering of cases was performed with the two-step automatic clustering method, which considered all symptoms with an observed frequency of at least 5% plus the following five clinical and demographic variables: sex, presence of comorbidities, phase of the pandemic (Omicron vs. pre-Omicron), number of symptoms, and WHO grade of severity of acute disease (mild vs. moderate/severe), with the final number of clusters automatically selected according to the quality of the model provided by the Schwarz Bayesian information criterion.

Potential risk factors independently associated with persisting symptoms at second evaluation were evaluated in a multivariable logistic regression model that included age, sex, number of symptoms at first evaluation, phase of acute disease (Omicron vs. pre-Omicron), presence of comorbidities, and severity of acute disease (mild vs. moderate/severe) as independent variables. All analyses were performed using the SPSS software, version 27.0 (IBM Corp., 2017, Armonk, NY, USA).

## 3. Results

### 3.1. Clinical Characteristics of Adolescent Patients Accessing Care for Long-COVID

The study population included 97 adolescent patients aged 12–17 years (53 females, 44 males), evaluated at three clinical centres after a mean interval from acute infection of 96.5 days (SD 52). Their general characteristics are shown in Table 1. No significant differences were observed between males and females in terms of age, vaccination status, epidemic phase of acute infection, or severity of acute infection (mild in 90.7% of cases). Only three cases were hospitalised during acute infection and none required respiratory assistance. Eight adolescents (8.2%) had relevant comorbidities (three had asthma, five had other conditions) (Table 1).

### 3.2. Complications Related to Acute SARS-CoV-2 Infection

Nine patients (9.3%) following acute SARS-CoV-2 infection were diagnosed with new conditions that were considered related to COVID: pericarditis (2), low blood pressure (1), pancreatitis (1), sleep disturbances (1), anxiety, depression, psychosis, post-traumatic stress disorder (1), polyneuropathy (1), fasciculations (1), and asthma (1). Most of these conditions occurred in the first few weeks after acute infection and all resulted to be resolved at follow up evaluation.

### 3.3. Profile of Symptoms Reported and Variables Associated with Symptoms

The mean number of symptoms reported by the entire adolescent population was 2.8 (SD 1.8), with no significant differences by sex (3.0 in females vs. 2.7 in males, *p* = 0.526), vaccination status (2.8 in vaccinated vs. 2.9 in unvaccinated, *p* = 0.658) or presence of comorbidities (2.5 with comorbidities vs. 2.9 without comorbidities, *p* = 0.563), but with significant differences by pandemic phase (3.2 for pre-Omicron vs. 2.5 for Omicron, *p* = 0.046) and by severity of acute disease (4.2 in patients with moderate or severe acute disease compared to 2.7 in those with mild acute disease, *p* = 0.023).

The observed symptom profile included 23 different symptoms. None of the adolescent reported as persistent symptoms paresthesia, weight loss, disorders of equilibrium or gait, hearing disturbances, pharyngodynia, chilblains, delirium, or hallucinations. The prevalence of the 13 symptoms more commonly reported (at least five per cent of cases) were compared by sex, phase of the pandemic, presence of comorbidities, and severity of the acute disease. No significant differences were observed by sex (Supplementary Table S1). With respect to the phase of the pandemic, fatigue was significantly more common among adolescents infected during the Omicron phase compared to those infected during the pre-Omicron phase, (72.5% vs. 52.2%, *p* = 0.038). An opposite trend approaching statistical significance was observed for dyspnea and thoracic pain that were more common among those infected during the pre-Omicron phase compared to the Omicron phase (52.2% vs. 35.3% for dyspnea, *p* = 0.094, and 30.4% vs. 15.7% for thoracic pain, *p* = 0.083). With respect to comorbidities, fatigue was significantly more common among the adolescents without comorbidities (66.3% vs. 25.0% in those with comorbidities, *p* = 0.049), while thoracic pain was significantly more common among those with previous comorbidities (62.5% compared to 19.1% in those without comorbidities, *p* = 0.014) (Table 2).

Finally, with respect to severity of acute disease, palpitations/tachycardia were significantly more common among adolescents with more severe acute disease (50.0% for moderate or severe acute disease compared to 14.8% for mild disease, *p* = 0.031), with a similar trend approaching statistical significance for muscular pain (37.5% vs. 10.2%, *p* = 0.059), fever (25.0% vs. 4.5%, *p* = 0.077), articular pain (37.5% vs. 13.6%, *p* = 0.107), and thoracic pain (50.0% vs. 19.3%, *p* = 0.066) (Table 2).

**Figure 1:**
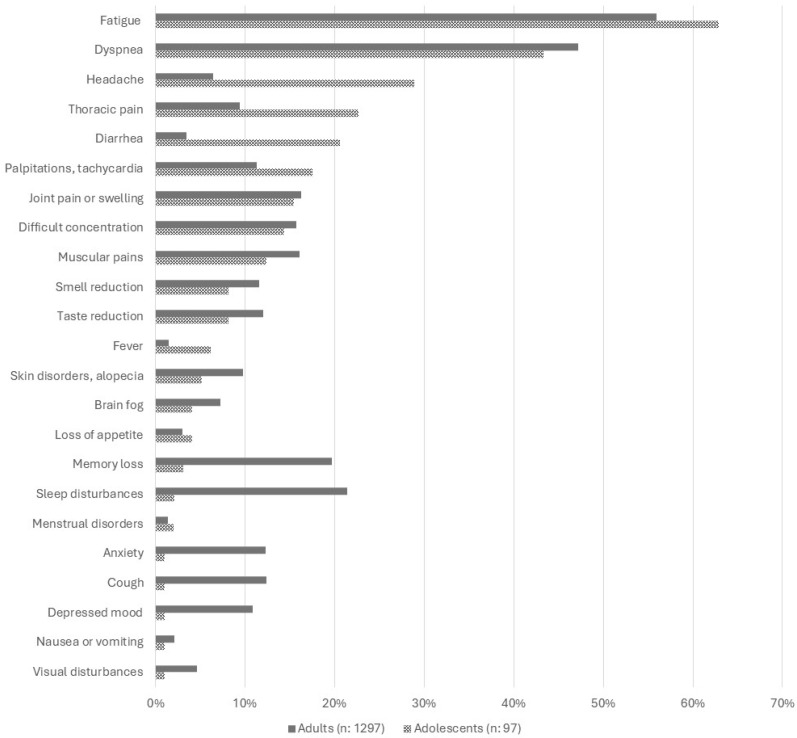
Prevalence of persisting symptoms in contemporarily enrolled adults and adolescents.

Overall, the symptom profile was markedly different from that observed in 1297 adults contemporarily enrolled in the same project, with twelve symptoms showing significant differences between the two age groups: headache, thoracic pain, diarrhea, and fever were significantly more frequent in adolescents; while memory loss, sleep disturbances, depressed mood, cough, anxiety, weight loss, paresthesias, and gait/equilibrium disturbances were significantly more common in adults (Figure 1 and Supplementary Table S2).

### 3.4. Clustering of Cases

The cluster analysis automatically selected three- and two-cluster models as solutions with adequate statistical quality. Given the low number of cases, the two-cluster solution was preferred. The results are presented in Table 3. The two clusters were similar with respect to sex and presence of comorbidities, but cluster 1 was characterised by a lower prevalence of the more recent (Omicron) acute infection and by a higher overall number of symptoms that translated to a higher prevalence of most of the individual symptoms evaluated (Table 3).

### 3.5. Diagnostic Tests

The clinical evaluation and the diagnosis of new conditions was accompanied by a variety of instrumental tests that most commonly included chest ultrasonography (42, 43.3%), heart ultrasonography (17, 17.5%), electrocardiogram (9, 9.3%), cardiopulmonary exercise testing (9, 9.3%), Holter electrocardiography (9, 9.3%), and chest X-ray (5, 5.2%). The complete list of the instrumental tests performed is reported in Supplementary Table S3.

### 3.6. Symptom Persistence During Follow Up

**Figure 2:**
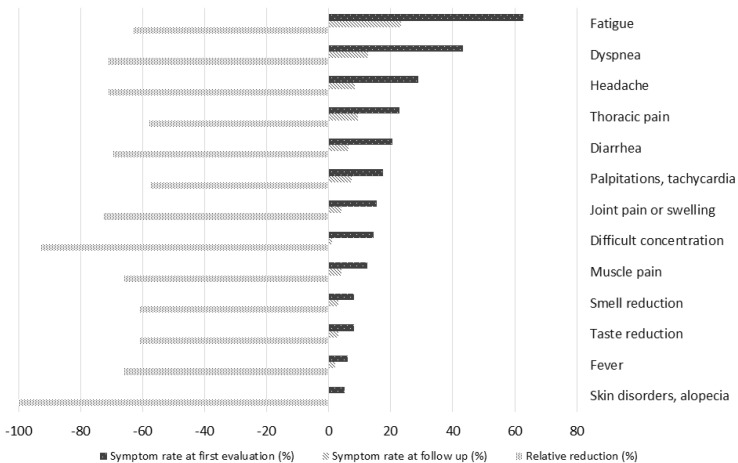
Follow up prevalence and relative reduction of the symptoms most commonly reported at first evaluation.

Ninety-five adolescents (97.9%) following first clinical long-COVID evaluation had a follow-up evaluation, performed after a mean interval from the first evaluation of 248 days (SD 94) and after a mean interval from acute infection of 340 days (SD 94). At this timepoint, 43 adolescents (45.3%) still presented with persistent symptoms. In this population of adolescents who remained symptomatic, the mean number of persistent symptoms decreased significantly from 3.3 (SD 1.9) to 2.1 (SD 1.3) (*p* < 0.001). No patients increased their symptom number compared to first evaluation. Seven symptoms (anxiety, depressed mood, sleeping disturbances, visual disturbances, loss of appetite, skin disorders, and menstrual disorders) that were present at the first evaluation in one or more of the adolescents were not reported at the second evaluation. The follow up rates of the most common initial symptoms and their proportional reduction compared to the first evaluation are reported in Figure 2.

The proportion of adolescents free of symptoms at the second evaluation was inversely correlated to the number of initial symptoms, with a significant decrease at the increasing of initial symptoms (68.0% in those with one symptom, 64.3% with two symptoms, 58.3% with three symptoms, 35.7% with four symptoms, 33.3% with five symptoms, and 42.9% with 6 symptoms) (*p* = 0.025, chi square for trend for the inverse correlation).

Finally, we assessed the potential risk factors independently associated with persisting symptoms at the second evaluation, considering age, sex, number of symptoms at first evaluation, phase of acute disease (Omicron vs. pre-Omicron), presence of comorbidities, and severity of acute disease (mild vs. moderate/severe) as independent variables. In the corresponding multivariable logistic regression model, only the number of symptoms at the first evaluation was significantly associated with persisting symptoms (adjusted odds ratio per each additional symptom reported: 1.354, 95%CI 1.022–1.794, *p* = 0.035). The full results of the multivariable logistic regression model are shown in Supplementary Table S4.

## 4. Discussion

The present study characterised a population of 97 adolescents accessing care for persistent symptoms following SARS-CoV-2 infection. The mean number of symptoms did not show differences by sex or vaccination status but was significantly higher for infections that occurred in the pre-Omicron phase and for patients with the more severe acute disease. This finding is consistent with several studies that have linked the presence and persistence of post-COVID conditions to a more severe acute disease, possibly driven by the more virulent variants circulating in the earlier phases of the pandemic [8,17].

Fatigue (62.9%) and dyspnea (43.3%) were the most commonly observed symptoms. Such rates are consistent with those that we observed in contemporarily enrolled adults (55.9% and 47.2%, respectively) [21]. Fatigue is consistently reported by several studies as the most common long-COVID manifestation in children and adolescents [12,13,15,16], and has been recognised as a predominant factor affecting quality of life in paediatric long-COVID [22]. It has also been included as a critical outcome in the Core Outcome measurement set produced by the International PC-COS Children Consensus [23] and as a key symptom in the WHO consensus on Post-COVID clinical case definition in children and adolescents [7]. In our study, its prevalence reduced three-fold during follow up, and no cases of its most severe form, represented by chronic fatigue syndrome/myalgic encephalomyelitis (CFS/ME), were diagnosed. Despite these favourable findings, fatigue remained, even after a longer follow up, the most common persistent symptom, with a final prevalence in our case series of roughly 20% that deserves clinical attention, particularly because this symptom appeared to be significantly more common among adolescents more recently infected, suggesting a current clinical relevance of this finding.

Dyspnea was the second most frequent symptom. The prevalence rates reported for this symptom in published studies of paediatric and adolescent individuals are highly variable, with some studies reporting it as a key symptom [11,16,17], while other studies found it to be much less frequent compared to other manifestations [9,12,13,15]. Although such differences may be explained by variable definitions, settings, and populations, persistent dyspnea might also reflect a more severe involvement of the respiratory system in acute SARS-CoV-2 disease, possibly due to the more aggressive variants circulating during the early pandemic [8,24]. Consistent with this assumption, in our cluster analysis, all cases of dyspnea aggregated in cluster one, predominantly composed by adolescents infected during the pre-Omicron phase, and no cases were present in cluster 2, where almost all individuals were infected during the Omicron phase.

Headache, thoracic pain, and diarrhea were also frequent symptoms, affecting more than 20% of cases. These three symptoms were much less frequent in the adult population concurrently studied (6.4%, 9.4%, and 3.5%, respectively), and characterised together with fever (6.2% vs. 1.5% in adults), the specific symptom profile of adolescents, confirming the different clinical phenotypes of long-COVID already described by systematic reviews, multicentre studies, and consensus statements [7,11]. Based on these findings, we suggest that, in the evaluation of adolescents for long-COVID, in addition to fatigue, dyspnea, palpitations, difficult concentration, articular or muscle pain, smell or taste changes that are equally common in young and older age, particular attention should be paid to these symptoms that appear to be more frequent in younger patients and that can significantly affect quality of life and daily living [7].

The cluster analysis indirectly confirmed the association between pre-Omicron infection and a higher number of symptoms (3.5 vs. 1.5 in clusters with pre-Omicron and Omicron predominance, respectively). Although the results for individual symptoms, as already discussed, were particularly striking for dyspnea, that was present only in cluster one, most of the other symptoms were significantly more frequent in cluster one, with the exception of fatigue, headache, and fever, that were more frequent, although not significantly, in cluster two. Overall, these findings suggest that the acute infections occurred during the Omicron phase, while generally milder, may translate in a different long-COVID phenotype, possibly through distinct pathogenetic pathways that should be explored. In general, the few studies that have evaluated the clinical phenotypes of long-COVID in children and adolescents have provided inconsistent results [25,26,27]. Further research on this issue is needed.

The diagnostic tests that accompanied the clinical evaluation were most commonly directed to respiratory and cardiac morbidities. In this regard, some centres are offering advanced diagnostics for these patients to measure and monitor fatigue and inappropriate tachycardia syndromes, like cardiopulmonary exercise testing and 24-h ECG with measurement of heart rate variability [28]. Following acute infection, roughly ten per cent of adolescents had COVID-19 related clinical conditions diagnosed, usually in the first few weeks after acute infection, and all such conditions were no more present during follow up, providing reassurance on their reversibility.

The follow up evaluation showed that, after a mean interval of eight months from initial visit and at one year from acute infection, more than half of the adolescents who were previously symptomatic were symptom-free. Moreover, among those who remained symptomatic, the mean number of symptoms decreased significantly. Such findings are consistent with other studies that showed improvement in recovery rates at the increase of follow up [8,16]. Despite such reassuring findings, a significant proportion of adolescents remained symptomatic at one year from acute infection, and a longer follow up is essential to better define the long-term trajectories of symptoms. The association between persistence and the initial number of symptoms, confirmed in a multivariable analysis that was adjusted for age, sex, phase, and severity of acute disease, indicates that initial polysymptomaticity should be considered a risk factor for longer persistence.

The strengths of the present study include the evaluation of a population strictly characterised by precise age limits, while other studies have usually included mixed populations of children and adolescents, who may differ in several aspects. We also were able to show that the symptom profile of adolescents presents some significant differences with that of adults who were concurrently evaluated with the same instrument. The use of a WHO instrument may also allow comparisons with other international studies. We were also able to evaluate, together with a large number of symptoms, several demographic and clinical characteristics, such as comorbidities, severity of acute disease, and phase of the pandemic, assessing their potential role on the number and type of symptoms.

In terms of study limitations, our study was based on patients accessing care for long-COVID symptoms and did not allow any estimate of the prevalence of this condition. Having focused on patients evaluated in specialised centres, we also may have selected cases characterised by greater severity or complexity. We also did not evaluate psychosocial factors, measures of familiar well-being, quality of life, or impact on daily life and on educational attendance and performance, that would have usefully complemented the data collected [22,23,29].

Our data also do not allow for any conclusions about the possible role of vaccination in preventing long-COVID; this has been analysed by some authors, with variable results [15,30,31,32]. In our study, the proportion of adolescents vaccinated before infection was low. This might indirectly suggest a protective effect of vaccination in preventing long-COVID, but specifically designed prospective studies are needed to address this issue. With respect to symptoms, our instrument did not include some of the symptoms that were highlighted by the WHO consensus, issued in February 2023, while our study was already ongoing [7]. Finally, the identification of possible predictors of the persistence of individual symptoms was limited by the size of the sample studied.

## 5. Conclusions

In conclusion, this study characterised the symptom profile of a population only composed of adolescents that accessed care for persistent symptoms following SARS-CoV-2 infection. The findings indicate that, although the main symptoms, represented by fatigue and dyspnea, are common to adolescent and adult individuals, headache, thoracic pain, diarrhea, and fever are significantly more common in adolescents, defining a specific long-COVID phenotype for this age group. The phase of the pandemic, possibly through earlier variants determining increased disease severity, influenced the number and the type of symptoms. More than half of the previously symptomatic adolescents were symptom-free at one year, with a better outcome than those who had fewer initial symptoms. The above findings, contributing to a further definition of the possible manifestations of this condition and of its clinical correlates in adolescents, may be of interest for paediatricians, epidemiologists, and public health officers and could provide the basis for future clinical studies.

## Figures and Tables

**Table 1:** Population characteristics.

	All	Female	Male	*p*
Sex: (n, %)	97 (100%)	53 (54.6%)	44 (45.4%)	
Age (years, mean, SD)	13.5 (1.5)	13.4 (1.5)	13.6 (1.6)	0.502
Comorbidities (n, %)	8 (8.2%)	4 (7.5%)	4 (9.1%)	0.783
Asthma	3	3	0	
Tourette syndrome, anxiety	1	0	1	
Obesity, pituitary hypoplasia, metabolic syndrome, anxiety	1	0	1	
Hashimoto thyroiditis	1	0	1	
Epilepsy	1	0	1	
Trisomy 21	1	1	0	
Vaccinated before infection *	40 (43.0%)	21 (42.0%)	19 (44.2%)	0.832
Not vaccinated	53 (57.0%)	29 (58.0%)	24 (55.8%)	
Acute infection pandemic phase: Pre-Omicron	46 (47.4%)	25 (47.2%)	21 (47.7%)	0.956
Omicron	51 (52.6%)	28 (52.8%)	23 (52.3%)	
Hospitalised during acute phase (n: 96):	3 (3.1%)	1 (1.9%)	2 (4.5%)	0.462
Admitted to intensive care unit:	0	0	0	
WHO COVID severity grade: Mild	88 (90.7%)	49 (92.5%)	39 (88.6%)	0.482
Moderate	7 (7.2%)	3 (5.7%)	4 (9.1%)	
Severe	1 (1.0%)	0 (0%)	1 (2.3%)	
Critical	0	0	0	
Unknown	1 (1.0%)	1 (1.9%)	0 (0%)	
Respiratory assistance: None	96 (99.0%)	52 (98.1%)	44 (100%)	0.360
Unknown	1 (1.0%)	1 (1.9%)	0 (0%)	

SD: standard deviation; * N with known vaccination status = 93. Vaccinated: any dose.

**Table 2:** Symptom prevalence by phase of the pandemic, presence of comorbidities and severity of acute disease.

		Phase of Pandemic			Comorbidities			Severity of Acute Disease		
	All (n: 97)	Omicron (n: 51)	Pre-Omicron (n: 46)	*p* Value	Yes (n: 8)	No (n: 89)	*p* Value	Mild (n: 88)	Moderate/Severe (n: 8)	*p* Value
Fatigue (n, %)	61 (62.9%)	37 (72.5)	24 (52.2)	0.038	2 (25.0)	59 (66.3)	0.049	54 (61.4)	6 (75.0)	0.706
Dyspnea (n, %)	42 (43.3%)	18 (35.3)	24 (52.2)	0.094	4 (50.0)	38 (42.7)	0.724	37 (42.0)	5 (62.5)	0.292
Headache (n, %)	28 (28.9%)	16 (31.4)	12 (26.1)	0.566	2 (25.0)	26 (29.2)	1.000	25 (28.4)	3 (37.5)	0.688
Thoracic pain (n, %)	22 (22.7%)	8 (15.7)	14 (30.4)	0.083	5 (62.5)	17 (19.1)	0.014	17 (19.3)	4 (50.0)	0.066
Diarrhea (n, %)	20 (20.6%)	8 (15.7)	12 (26.1)	0.206	1 (12.5)	19 (21.3)	1.000	19 (21.6)	1 (12.5)	1.000
Palpitations, tachycardia (n, %)	17 (17.5%)	8 (15.7)	9 (19.6)	0.616	1 (12.5)	16 (18.0)	1.000	13 (14.8)	4 (50.0)	0.031
Joint pain or swelling (n, %)	15 (15.5%)	7 (13.7)	8 17.4)	0.618	1 (12.5)	14 (15.7)	1.000	12 (13.6)	3 (37.5)	0.107
Difficult concentration (n, %)	14 (14.4%)	6 (11.8)	8 (17.4)	0.431	1 (12.5)	13 (14.6)	1.000	13 (14.8)	1 (12.5)	1.000
Muscle pain (n, %)	12 (12.4%)	5 (9.8)	7 (15.2)	0.419	0 (0)	12 (13.5)	0.590	9 (10.2)	3 (37.5)	0.059
Taste reduction (n, %)	8 (8.2%)	2 (3.9)	6 (13.0)	0.145	0 (0)	8 (9.0)	1.000	8 (9.1)	0 (0)	1.000
Smell reduction (n, %)	8 (8.2%)	2 (3.9)	6 (13.0)	0.145	1 (12.5)	7 (7.9)	0.511	8 (9.1)	0 (0)	1.000
Fever (n, %)	6 (6.2%)	1 (2.2)	5 (9.8)	0.208	1 (12.5)	5 (5.6)	0.412	4 (4.5)	2 (25.0)	0.077
Skin disorders, alopecia (n, %)	5 (5.2%)	3 (5.9)	2 (4.3)	1.000	1 (12.5)	4 (4.5)	0.356	5 (5.7)	0 (0)	1.000

Other less frequently reported symptoms included brain fog (4 cases), loss of appetite (4 cases), memory loss (3 cases), sleep disturbances (2 cases), menstrual disorders (1 case), visual disturbances (1 case), nausea or vomiting (1 case), depressed mood (1 case), cough (1 case), and anxiety (1 case).

**Table 3:** Case clustering by clinical and demographic variables and symptoms reported.

	Cluster 1	Cluster 2	*p*
N (96)	64	32	
Female (n, %)	38 (59.4)	14 (43.8)	0.148
Omicron phase of the pandemic (n, %)	22 (34.4)	28 (87.5)	<0.001
Presence of comorbidities (n, %)	5 (7.8)	3 (9.4)	1.000
Acute infection moderate or severe (n, %)	7 (10.9)	1 (3.1)	0.262
Number of symptoms reported (mean, SD)	3.5 (1.8)	1.5 (0.8)	<0.001
Fatigue (n, %)	38 (59.4)	22 (68.8)	0.371
Dyspnea (n, %)	42 (65.6)	0 (0)	<0.001
Headache (n, %)	16 (25.0)	12 (37.5)	0.204
Thoracic pain (n, %)	18 (28.1)	3 (9.4)	0.040
Diarrhea (n, %)	19 (29.7)	1 (3.1)	0.003
Palpitations, tachycardia (n, %)	15 (23.4)	2 (6.3)	0.047
Joint pain or swelling (n, %)	15 (23.4)	0 (0)	0.002
Difficult concentration (n, %)	13 (20.3)	1 (3.1)	0.030
Muscle pain (n, %)	12 (18.8)	0 (0)	0.007
Taste reduction (n, %)	8 (12.5)	0 (0)	0.049
Smell reduction (n, %)	8 (12.5)	0 (0)	0.049
Fever (n, %)	2 (3.1)	4 (12.5)	0.093
Skin disorders, alopecia (n, %)	4 (6.3)	1 (3.1)	0.662

## Data Availability

The study data can be made available upon reasonable request. Ethics Committee consultation may be necessary in order to obtain permission to share. Requests to access the datasets should be directed to marco.floridia@iss.it.

## Supplementary Materials

The following supporting information can be downloaded at: https://www.mdpi.com/article/10.3390/children12010028/s1, Table S1: Symptom prevalence by sex; Table S2: Symptom profile in contemporarily enrolled adolescent and adults; Table S3: Instrumental tests performed; Table S4: Multivariable analysis of factors associated with persisting symptoms during follow-up.
