# Supplementary material for: Adolescents with Persistent Symptoms Following Acute SARS-CoV-2 Infection (Long-COVID): Symptom Profile, Clustering and Follow-Up Symptom Evaluation
Source: Children (Basel). 2024 Dec 27;12(1):28. doi: 10.3390/children12010028 (PMC11763728; doi:10.3390/children12010028)
Supplement: Supplementary file 1 [file children-12-00028-s001.zip › children-3341235-supplementary.pdf]

Supplementary Table S1. Symptom profile in contemporarily enrolled adolescent and adults.

	Adolescents (n: 97)	Adults (n: 1297)	p
Fatigue	61 (62.9%)	725 (55.9%)	0.181
Dyspnea	42 (43.3%)	612 (47.2%)	0.459
Headache	28 (28.9%)	83 (6.4%)	**<0.001**
Thoracic pain	22 (22.7%)	122 (9.4%)	**<0.001**
Diarrhea	20 (20.6%)	45 (3.5%)	**<0.001**
Palpitations, tachycardia	17 (17.5%)	146 (11.3%)	0.064
Joint pain or swelling	15 (15.5%)	212 (16.3%)	0.821
Difficult concentration	14 (14.4%)	204 (15.7%)	0.735
Muscular pains	12 (12.4%)	209 (16.1%)	0.330
Taste reduction	8 (8.2%)	155 (12.0%)	0.274
Smell reduction	8 (8.2%)	151 (11.6%)	0.310
Fever	6 (6.2%)	19 (1.5%)	**<0.001**
Skin disorders, alopecia	5 (5.2%)	127 (9.8%)	0.132
Loss of appetite	4 (4.1%)	39 (3.0%)	0.536
Brain fog	4 (4.1%)	95 (7.3%)	0.164
Memory loss	3 (3.1%)	255 (19.7%)	**<0.001**
Sleep disturbances	2 (2.1%)	277 (21.4%)	**<0.001**
Menstrual disorders	1 (2.0%)	9 (1.4%)	0.515
Visual disturbances	1 (1.0%)	60 (4.6%)	0.120
Nausea or vomiting	1 (1.0%)	27 (2.1%)	0.716
Depressed mood	1 (1.0%)	142 (10.9%)	**<0.001**
Cough	1 (1.0%)	161 (12.4%)	**<0.001**
Anxiety	1 (1.0%)	160 (12.3%)	**< 0.001**
Weight loss	0 (0%)	53 (4.1%)	**0.047**
Chilbains	0 (0%)	8 (0.6%)	1.000
Paresthesias	0 (0%)	113 (8.7%)	**<0.001**
Delirium, allucinations	0 (0%)	3 (0.2%)	1.000
Disorders of equilibrium or gait	0 (0%)	71 (5.5%)	0.008
Hearing disorders	0 (0%)	41 (3.2%)	0.110
Pharingodinia	0 (0%)	41 (3.2%)	0.110

Supplementary Table S2. Instrumental tests performed.

Chest Ultrasonography	42 (43.3)
Heart ultrasonography	17 (17.5)
Electrocardiography	9 (9.3%)
Cardiopulmonary exercise testing	9 (9.3)
Holter electrocardiography	7 (7.2)
Chest X-ray	5 (5.2)
Lung single photon emission computed tomography	2 (2.1)
Heart magnetic resonance imaging	2 (2.1)
Abdominal ultrasonography	2 (2.1)
Brain single photon emission computed tomography	1 (1.0)
Brain magnetic resonance imaging	1 (1.0)
Chest computed tomography scan	1 (1.0)
Arteriovenous Doppler ultrasonography	1 (1.0%)

Supplementary Table S3. Instrumental tests performed.

Chest Ultrasonography	42 (43.3)
Heart ultrasonography	17 (17.5)
Electrocardiography	9 (9.3%)
Cardiopulmonary exercise testing	9 (9.3)
Holter electrocardiography	7 (7.2)
Chest X-ray	5 (5.2)
Lung single photon emission computed tomography	2 (2.1)
Heart magnetic resonance imaging	2 (2.1)
Abdominal ultrasonography	2 (2.1)
Brain single photon emission computed tomography	1 (1.0)
Brain magnetic resonance imaging	1 (1.0)
Chest computed tomography scan	1 (1.0)
Arteriovenous Doppler ultrasonography	1 (1.0%)

Supplementary Table S4. Multivariable analysis of factors associated with persisting symptoms during follow-up.

	Adjusted odds ratio	95% CI	P
Male sex	1.218	0.493-3.007	0.669
Age	1.150	0.850-1.556	0.364
Pre-Omicron phase of infection	2.047	0.833-5.031	0.118
Moderate or severe acute disease	3.915	0.406-37.77	0.238
Presence of comorbidities	1.786	0.334-9.536	0.498
Number of symptoms at first evaluation	1.354	1.022-1.794	**0.035**

Multivariable logistic regression model based on 94 cases (96.9%). Independent variables were age, sex, number of symptoms at first evaluation, phase of acute disease (Pre-Omicron vs. Omicron), presence of comorbidities, and severity of acute disease (mild vs. moderate/severe). CI: Confidence interval.
